# The Persimmon 9-lipoxygenase Gene *DkLOX3* Plays Positive Roles in Both Promoting Senescence and Enhancing Tolerance to Abiotic Stress

**DOI:** 10.3389/fpls.2015.01073

**Published:** 2015-12-10

**Authors:** Yali Hou, Kun Meng, Ye Han, Qiuyan Ban, Biao Wang, Jiangtao Suo, Jingyi Lv, Jingping Rao

**Affiliations:** ^1^State Key Laboratory of Crop Stress Biology for Arid Areas, College of Horticulture, Northwest A&F UniversityYangling, China; ^2^College of Horticulture, Shenyang Agricultural UniversityShenyang, China

**Keywords:** persimmon, 9-lipoxygenase, fruit ripening, softening, senescence, abiotic stress

## Abstract

The lipoxygenase (LOX) pathway is a key regulator for lipid peroxidation, which is crucial for plant senescence and defense pathways. In this study, the transcriptional expression patterns of three persimmon (*Diospyros kaki* L. ‘Fupingjianshi’) 9-lipoxygenase genes (*DkLOX1*, *DkLOX3*, and *DkLOX4*) were investigated. *DkLOX1* was specifically expressed in fruit, particularly in young fruit, and showed little response to the postharvest environments. *DkLOX4* was expressed in all tissues and slightly stimulated by mechanical damage and low temperature. *DkLOX3* was expressed mainly in mature fruit, and the expression was extremely high throughout the storage period, apparently up-regulated by mechanical damage and high carbon dioxide treatments. Further functional analysis showed that overexpression of *DkLOX3* in tomato (*Solanum lycopersicum* cv. Micro-Tom) accelerated fruit ripening and softening. This was accompanied by higher malondialdehyde (MDA) content and lycopene accumulation, advanced ethylene release peak and elevated expression of ethylene synthesis genes, including *ACS2*, *ACO1*, and *ACO3*. In addition, *DkLOX3* overexpression promoted dark induced transgenic *Arabidopsis* leaf senescence with more chlorophyll loss, increased electrolyte leakage and MDA content. Furthermore, the functions of *DkLOX3* in response to abiotic stresses, including osmotic stress, high salinity and drought were investigated. *Arabidopsis DkLOX3* overexpression (*DkLOX3*-OX) transgenic lines were found to be more tolerant to osmotic stress with higher germination rate and root growth than wild-type. Moreover, *DkLOX3*-OX *Arabidopsis* plants also exhibited enhanced resistance to high salinity and drought, with similar decreased O_2_^-^ and H_2_O_2_ accumulation and upregulation of stress-responsive genes expression, including *RD22*, *RD29A*, *RD29B*, and *NCED3*, except for *FRY1*, which plays a negative role in stress response. Overall, these results suggested that *DkLOX3* plays positive roles both in promoting ripening and senescence through lipid peroxidation and accelerated ethylene production and in stress response via regulating reactive oxygen species accumulation and stress responsive genes expression.

## Introduction

Persimmon (*Diospyros kaki* L.) is a high-economic-value crop that is rich in a variety of nutrients and mineral substances, such as Na, K, Ca, Mg, Fe, and Mn in whole persimmons, their pulps and peels (Gorinstein et al., 2001; Kausch et al., 2012). However, as a kind of climacteric fruit, persimmons soften and decay quickly after harvest, thereby affecting their marketability (Zhang et al., 2012).

Fruit ripening and senescence is the summation of many biochemical and physiological changes (Giovannoni, 2001). Lipoxygenases (LOXs) has long been considered to associate with fruit ripening and senescence (Zhang et al., 2006). In previous studies, lipoxygenase has shown higher enzyme activity accompanied by more serious lipid peroxidation, and elevated *LOX* genes expression in senescent tissues, including *Arabidopsis* leaves (He et al., 2002), broccoli (*Brassica oleracea*; Eugenia Gomez-Lobato et al., 2012), or ripening fruit such as tomato (*Solanum lycopersicum*; Heitz et al., 1997; Griffiths et al., 1999), pear (*Prunus persica*; Han et al., 2011), kiwifruit (*Actinidia chinensis*; Zhang et al., 2006), cucumber (*Cucumis sativus*; Yang et al., 2012), and melon (*Cucumis melo*; Zhang et al., 2014). The mechanisms involved were assumed to be associated with lipid peroxidation catalyzed by LOX, which causes membrane deterioration (Rogiers et al., 1998; Zhang et al., 2006). Moreover, LOX-derived reactive oxygen species (ROS) production also likely contributes to plant ripening and senescence (Pilati et al., 2014).

In climacteric fruit, ethylene is necessary for the coordination and completion of ripening (Giovannoni, 2001), accompanied by a peak of respiration and a concomitant burst of ethylene production (Alexander and Grierson, 2002). There were considerable differences among the *LOX* genes in expression patterns during fruit ripening and these patterns were divided into two groups (Zhang et al., 2006). The transcript levels of one group were generally decreased as fruit ripening and had little relationship to the LOX activity and malondialdehyde (MDA) content; kiwifruit *AdLOX2*, *AdLOX3*, *AdLOX4*, *AdLOX6*, tomato *TomLOXA*, and peach *PpLOX3* were classified into this group (Griffiths et al., 1999; Zhang et al., 2006; Han et al., 2011). The other group of the genes initially had negligible transcript levels at the early stage after harvest, but showed a response to external ethylene at the pre- and climacteric stages, which seems related to the increases in LOX activity and MDA content, such as *AdLOX1* and *AdLOX5*, *TomLOXB*, and *PpLOX1* (Griffiths et al., 1999; Zhang et al., 2006; Han et al., 2011). It is mainly the latter group of lipoxygenase, that might cooperate with ethylene and play a significant role in fruit maturation and senescence, but the detailed mechanism is remain unclear.

In addition to the important features of LOX action on cell membrane degradative processes occurring in fruit ripening, the products of lipid peroxidation, known as phyto-oxylipins, also have shown the important roles in signaling and plant defense responses (Porta and Rocha-Sosa, 2002). Plant LOXs can be categorized as either 9-LOXs or 13-LOXs depending on the position at which the oxygen is incorporated into linoleic acid or linolenic acid (Feussner and Wasternack, 2002). 13-LOX is involved in the generation of jasmonic acid (JA) and C5 or C6 volatile (Chen et al., 2004; Hwang and Hwang, 2010; Shen et al., 2014). JA is considered to be a key regulator for stress-induced gene expression to enhance resistance (Yan et al., 2013). There were many studies focusing mainly on oxylipins produced through 13-LOX pathway. Overexpression of tomato *LOXD* increased synthesis of JA and enhanced resistance to pathogenic fungus, high temperature (Hu et al., 2013), as well as insect attack and mechanical wounding (Yan et al., 2013). However, to date, the defense-related functions of 9-LOXs have been still poorly understood. It has been found that salt stress could significantly increase LOX activity in rice (Mostofa et al., 2015) and tomato (Shalata and Tal, 1998; Mittova et al., 2002). Drought stress could also significantly increase LOX activity in olive (Sofo et al., 2004) and brassica seedlings (Alam et al., 2014). In particular, salt stress could specifically induce 9-*LOX* expression in citrus (Ben-Hayyim et al., 2001).

In our previous study, three persimmon *LOX* genes, *DkLOX1* (JF436951), *DkLOX3* (KF035131), and *DkLOX4* (KF035132) were cloned and shown to belong to the 9-LOX sub-group based on phylogenetic analysis (Lv et al., 2014). In addition, our previous study found that persimmon *DkLOX1* and *DkLOX3* shown a response to ABA and GA_3_, especially, the expression level of *DkLOX3* was very high and peaked with the lipoxygenase enzyme activity and ethylene production, which indicated that *DkLOX3* might contributed to persimmon fruit ripening (Lv et al., 2014). Many other researches also demonstrated that the expression of *LOX* genes could be induced by senescence and other stresses, however, there has always been lack of direct genetic evidence. In this study, expression patterns of persimmon *LOX* genes during fruit development and postharvest softening were investigated, and the exact role of *DkLOX3* in fruit ripening and softening and leaf senescence was verified in overexpressed transgenic tomato fruit and *Arabidopsis* leaves. In addition, the responses of transgenic *Arabidopsis* to the abiotic stresses were also investigated.

## Materials and Methods

### Plant Materials

The persimmon ‘Fupingjianshi’ (*D. kaki*), an astringent cultivar, was used in the present study. Different tissues, leaves, flowers, calyces, and stems were collected from adult persimmon trees. Young persimmon fruits were sampled at 20, 40, 60, 80, 100, 120 days after full bloom, fruits were harvested for postharvest treatments at 150 days after full bloom. For postharvest softening and senescence analysis, fruits with uniform size and shape without visible defects were harvested with 70–80% surface yellow colouration from a commercial orchard in Fuping County, Shaanxi province of China.

*Arabidopsis thaliana* ecotype ‘Columbia’ (Col-0) and *S. lycopersicum* Mill. cultivar ‘Micro-Tom’ were used for the function analysis of *DkLOX3*.

**Table 1:** Storage conditions of persimmon fruit during postharvest treatments.

Treatment	Temperature	Concentration of O_2_ (%)	Concentration of CO_2_ (%)	Relative humidity (%)
Control	25°C	Normal	Normal	85–95
MD	25°C	Normal	Normal	85–95
HC	25°C	Normal	6–8	85–95
LO	25°C	2–3	Normal	85–95
CS	0 ± 1°C	Normal	Normal	85–95


### Post-harvest Treatments of Persimmon Fruits

Fruits without mechanical damage were selected and randomly divided into five groups, with 200 fruits in each, and were treated as described in **Table 1**. Fruits used for mechanical damage treatment were injured congruously by one peduncle.

Fruits were randomly chosen from subgroups every 4 days to determine the ethylene production and firmness. Fruits tissues were peeled and immediately frozen in liquid nitrogen and stored at -80°C until the MDA content, LOX activity and gene expression were analyzed.

### Fruit Firmness Measurement and Ethylene Production

Firmness was determined by a pressure tester (Model FT327, Effegi, Milan, Italy) for persimmon and penetrometer (Model FT02, Effegi, Milan, Italy) for Micro-Tom.

For ethylene production measurement, fruits were enclosed and sealed in a vacuum dryer at 25°C for 1 h, after that, 1 ml of gas was collected by a syringe three times. Ethylene production was determined by injecting a gas sample into a flame ionization detection GC-14A gas chromatograph (Shimadzu, Kyoto, Japan). The oven, detector and injector were operated at 70, 70, and 150°C, respectively, and the carrier gas (N_2_, H_2_, and air) flow rates were 0.5, 0.5, and 5 ml s^-1^, respectively.

### LOX Activity Assay and Determination of MDA Content

The LOX activity was measured according to Zhang et al. (2006), with slight modifications. Frozen samples were ground and homogenized in 10.0 ml of 0.1 M ice-cold sodium phosphate extraction buffer (pH 6.8), including 1% (v/v) Triton X-100 and 4% (w/v) polyvinylpyrrolidone (PVP). After being centrifuged, the supernatant was used for LOX enzyme activity determinations following the oxidation of linoleic acid (Sigma, USA) by measuring the absorbance at 234 nm using a UV-1800 spectrophotometry (Shimadzu, Kyoto, Japan). One unit of LOX activity was defined as a change in the absorbance of 0.01 min^-1^ using linoleic acid sodium salt as a substrate.

The MDA contents were measured according to Rogiers et al. (1998) with modifications. Samples were ground and homogenized in 5 ml of 10% (w/v) trichloroacetic acid (TCA), followed by centrifugation at 12,000 *g* for 15 min at 4°C. A mixture of 0.5 ml of the supernatant and 3.5 ml of 10% TCA containing 0.5% (w/v) thiobarbituric acid was incubated in boiling water bath for 15 min, subsequently cooled on ice and centrifuged at 12,000 *g* for 20 min at 4°C. The absorbances of supernatant at 450, 532 and 600 nm were determined by UV-1800 spectrophotometry.

### RNA Extraction and cDNA Synthesis

The total RNA of persimmon was isolated following the hot borate method (Wan and Wilkins, 1994). The total RNA of the *Arabidopsis* and tomato were extracted using a TransZol Up Plus RNA Kit (Transgen Biotech, Beijing, China). The first-strand cDNA was synthesized from 1 μg of RNA using the PrimeScriptTM RT Reagent Kit with the gDNA Eraser (Perfect Real Time; TaKaRa, Dalian, China) following the manufacturer’s protocol. The synthesized cDNA was diluted 10-fold for persimmon and tomato samples or 20-fold for *Arabidopsis* leaves for the following qPCR analysis.

### Expression Analysis by qPCR

qPCR was carried out according to the protocols described by the SYBR Premix Ex TaqTMII (TaKaRa, Dalian, China) on an iCycler iQ5 (Bio-Rad, USA). The PCR mixture was composed of 2 μl of diluted cDNA, 0.8 μl of each primer (10 μM), 6.4 μl of ddH_2_O and 10 μl of SYBR Premix Ex TaqTMII. The cycling conditions included an initial hot start at 95°C for 3 min, followed by 40 cycles of 95°C for 10 s, 58°C for 30 s with signal acquisition, 72°C for 20 s, then completed with a melting curve analysis program. *DkACTIN*, *AtACTIN2*, and *LeUBI3* were used as house-keeping genes for persimmon, *Arabidopsis* and tomato, respectively.

The primers that were used for quantitative RT-PCR are shown in **Supplementary Table S1**. The primers were designed using Primer Premier 5 software.

### Vector Construction and Generation of Transgenic Tomato

The persimmon *DkLOX3* gene contained a 2619-bp open reading frame encoding 873 amino acid and was inserted into the binary vector 35S:pVBG2307 which was developed from the manipulation of pBI121 and pBI221 (**Supplementary Figure S1**) (Ahmed et al., 2012). Then recombinant plasmid *DkLOX3*-pVBG2307 was introduced into *Agrobacterium tumefaciens* strain GV3101. Micro-Tom was transformed via *Agrobacterium*-mediated leaf transformation according the protocols of McCormick (1997) and Guo et al. (2012).

The seeds were sterilized and germinated on one-half MS solid medium containing 30 g/l sucrose (pH5.8) for 8–10 days, until the cotyledons were fully expanded with no or minimal true leaves. The ends of cotyledons were excised and the rest of the section was cut into two halves across the midvein on wet filter paper. The explants were placed upside down on pre-culture MS solid medium containing 30 g/l sucrose, 2.0 mg/l 6-BA, and 0.2 mg/l IAA (pH5.8) for 2 days, and then the explants were dipped into the bacterial suspension (*OD* = 0.5) with 100 mM/l acetosyringone and incubated for 10 min. After incubation, the explants were blotted dry and placed upside down on the pre-culture MS medium for co-cultivation for 2 days in the darkness. After 2 days’ co-cultivation, the explants were transferred to a selective MS solid medium containing 30 g/l sucrose, 2.0 mg/l 6-BA, 0.2 mg/l IAA, 100 mg/l kanamycin, and 500 mg/l carbenicillin (pH5.8), sub-cultured to induce callus and shoot formation. When shoots with true meristems were formed, the redundant cotyledons and callus were cut off and transferred to rooting MS solid medium containing 30 g/l sucrose, 0.02 mg/l IAA, 50 mg/l kanamycin and 500 mg/l carbenicillin (pH5.8) for 2 weeks. The rooting shoots were transferred into wet soil covered with plastic wrap. The plants obtained were PCR-confirmed to select positive transgenic lines (*T*_0_), and the seeds from *T*_0_ were collected for future research.

### Storage of Transgenic Micro-tom Fruits

Fruits of WT and *DkLOX3*-OX transgenic lines (OX-1, OX-3, OX-6) were harvested at mature green period when fruits were green and shiny with no obvious color change, stored at 25°C, 85–95% relative humidity. Every 3 days, firmness, ethylene production and color changes were determined, and the fruits tissues were frozen in liquid nitrogen for future research.

The color of the tomatoes were measured with a chroma meter CR-400 (Konica Minolta, Osaka, Japan) consisting of a head with an 8 mm diameter measuring area and a diffuse illumination/0° viewing. Readings are reported in the *L^∗^*, *a^∗^*, *b^∗^* system, while the *L^∗^*, *a^∗^* and *a^∗^/b^∗^* value showed a linear correlation with the ripening stages of the tomatoes (Arias et al., 2000).

### Dark-induced Senescence of *Arabidopsis*

*Arabidopsis* was transformed via the floral dip method (Clough and Bent, 1998). Homozygous *Arabidopsis* transgenic seeds (*T*_3_) were used for further research.

When *Arabidopsis* plants were grown in soil for 4 weeks, both detached leaves and the whole plants were used for inducing senescence in dark. Leaf numbers 5 and 6 were detached from rosettes, according to Sharabi-Schwager et al. (2010). The leaves were floated on water in 9-mm-diameter Petri dishes and stored for up to 4 days in the dark at 22°C to promote senescence. The whole plant were also placed in dark to induced senescence for 4 days, and then the degrees of leaves yellowing were counted, and leaves number 3 or 4 from transgenic and WT were used for histochemical staining (the details were shown in Detection of Reactive Oxygen Species and Cell Death). Detached leaves and plants stored in growth condition were served as control.

### Seeds Germination and Root Growth Assays of Transgenic *Arabidopsis* under Osmotic Stress

For the seed germination assays, 50–60 seeds from each line of transgenic and WT plants were sown on one-half MS medium or one-half MS medium that was supplemented with 130 mM NaCl or 200 mM mannitol. The percentage of germination was calculated based on the number of seeds with testa rupture at 8 days.

For root growth, the seeds were sown on one-half MS basal medium and grown for 4 days. Then, the seedlings were transferred and vertically placed onto one-half MS basal medium or one-half MS medium with 130 mM NaCl or 200 mM mannitol for another 10 days, before photographed and samples frozen in liquid nitrogen for further analysis.

### Salt and Drought Stresses Tests of Transgenic *Arabidopsis* Plants

For treatment of salt stress, 20 seeds from control and transgenic lines were sown in pots filled with soil (6 cm × 6 cm × 5.5 cm, 5 plants/pot). In order to observe the seedlings growth under salt stress, salt treatment started at the stage of 8 days old by irrigating 10 ml 400 mM NaCl solution (stress) or water (control) for each pot at 2-day intervals for 18 days. For histochemical staining and expression analysis of stress-responsive genes, 4-week-old plants were watered with 400 mM NaCl (ratio between volume of the soil in pot and volume of NaCl solution was 1:10), and leaves were sampled at 24 h after treatment.

For the drought resistance assay, 18-day-old plants were without water for 10 days, and then leaves were excised for histochemical staining and further analysis of stress-responsive genes expression. After the drought treatment, re-water was performed to recovery growth.

### Measurements of Chlorophyll and Electrolyte Leakage

Chlorophyll was extracted with 80% acetone overnight at 4°C, and the concentration was determined spectrophotometrically as described by Wellburn (1994).

Electrolyte leakage was measured according to McKersie et al. (1996) with slight modification. Ten disks (5 mm in diameter) were incubated in 20 ml ddH_2_O for 30 min, and the conductivities (*C*_1_) were determined by a conductivity meter (Model DDS 307, Inesa, Shanghai, China). Subsequently, the solution with disks was boiled for 15 min, and the conductivities (*C*_2_) were recorded. Electrolyte leakage was expressed as *C*_1_/*C*_2_.

### Detection of Reactive Oxygen Species and Cell Death

Leaves from control and salt or drought treated plants were excised for detecting O_2_^-^ and H_2_O_2_ accumulation. O_2_^-^ accumulation was detected according to Daudi and O’Brien (2012). Leaves were immersed in solution of 0.1% (w/v) nitro blue tetrazolium (NBT) in 10 mM potassium phosphate buffer (pH 7.8) and vacuum-infiltrated for 5 min followed by incubation at room temperature for 2 h in the dark. For detection of H_2_O_2_ accumulation, leaves were immersed in solution of 1 mg/ml diaminobenzidine (DAB), vacuum-infiltrated for 5 min and then incubated at room temperature for 8 h in the absence of light (Kumar et al., 2014). After staining with NBT or DAB, the leaves were distained with ethanol. To monitor cell death, leaves were stained with a boiled trypan blue solution [10 ml 85% (lactic acid), 10 g phenol, 10 ml glycerol, 10 mg trypan blue, and 10 ml ddH_2_O] for 5 min, and then washed with sterilized ddH_2_O and bleached with 2.5 g/ml chloral hydrate (Guo et al., 2015). After staining, samples were placed on filter paper and pictures were taken with reflected light by an Olympus BX-51 microscope (Olympus Corporation, Japan).

### Data Analysis

All of the experiments contained three biological replicates, and each replicate included at least three technical replicates. The results are represented as the mean ± standard errors. Significant differences among the means were assessed by analysis of one way ANOVA with the least significant difference (LSD) at *P* < 0.05 using SPSS 16.0 software.

## Results

### Firmness Changes, Ethylene Production, Lipoxygenase Activity and MDA Content during Postharvest Storage of Persimmon (*Diospyros kaki*)

**FIGURE 1:**
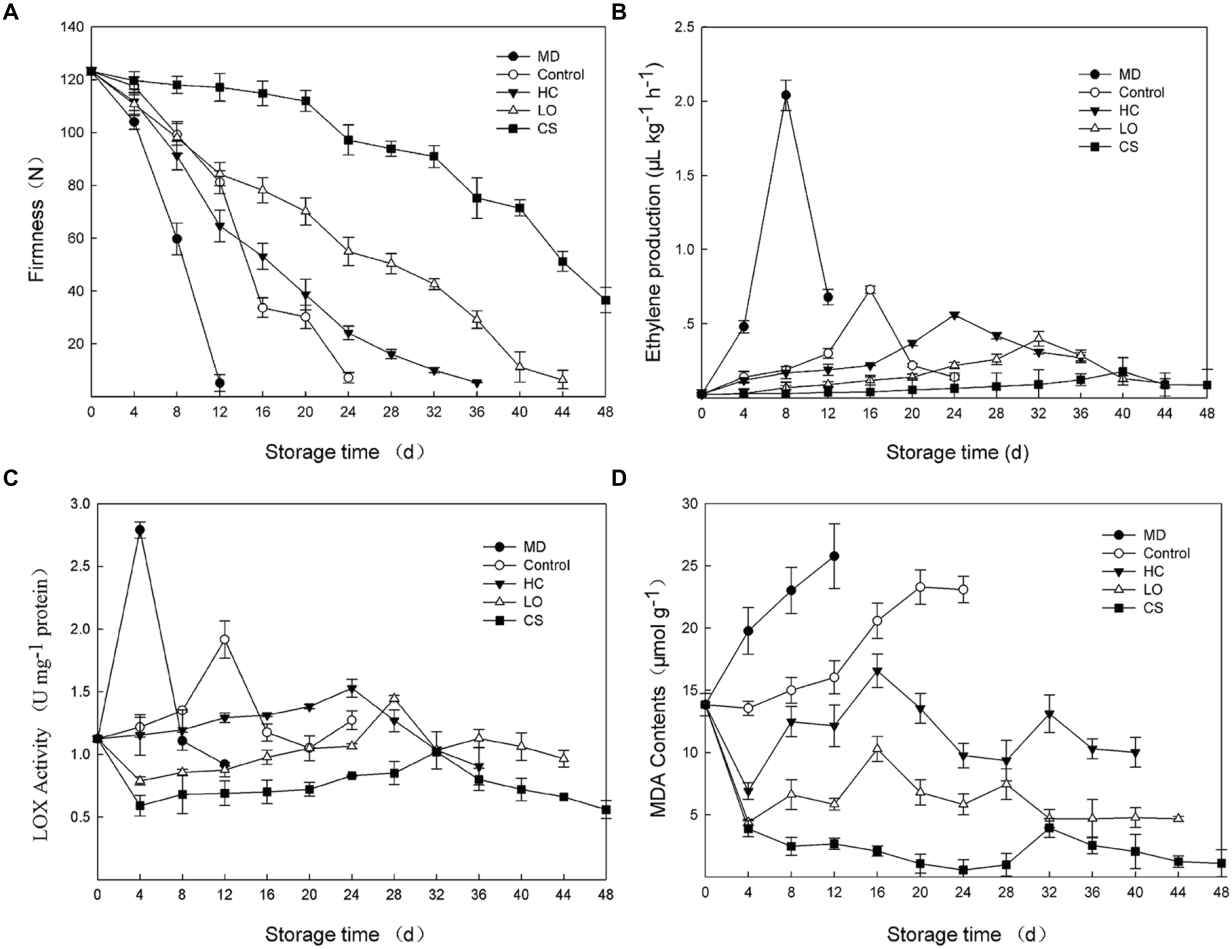
**Firmness changes **(A)**, ethylene production **(B)**, lipoxygenase activities **(C)** and malondialdehyde (MDA) contents **(D)** of persimmon fruits during storage following mechanical damage (MD, 25°C), high carbon dioxide (HC, 25°C, 6–8% CO_2_), low oxygen (LO, 25°C, 2–3% O_2_) and cold storage (CS, 4°C) conditions.** Untreated fruits stored at 25°C served as control. Data are presented as mean ± standard error from three replicates with three biological repeats, and different letters above the columns indicate significant difference (LSD, *P* < 0.05) between samples across the time-course of the experiment.

Ethylene production and tissue firmness are ripening indicators of fruit. With mechanical damage, fruits firmness decreased with the most rapid rate and was not detectable only 12 days (**Figure 1A**), meanwhile, ethylene production maintained at the highest level and reached a peak (2.04 μl kg^-1^ h^-1^) at 8 days, approximately 2.8-fold of the peak value of control fruits (**Figure 1B**). Fruit firmness could still be measured until 24, 36, 44, and 60 days with control, HC, LO, and CS treatments, respectively. For treatments with HC, LO, and CS, the firmness decreased slower (**Figure 1A**), while the peaks in ethylene production were delayed approximately 8–20 days compared to the control (**Figure 1B**).

The lipoxygenase activities of the mechanical damage and control fruits began to increase from the day of harvest and peaked (2.79 and 1.92 U/mg protein) on the 4 and 12th days, respectively (**Figure 1C**). However, the fruits that were treated with HC and LO displayed a slightly increase throughout the entire storage period, with a small peak on the 24 and 28th day, 12 and 16 days later than that observed in the control fruits, respectively (**Figure 1C**). CS treatment strongly suppressed the lipoxygenase activities of persimmon fruits (**Figure 1C**). MDA contents were measured as product of lipid peroxidation (Ayala et al., 2014). The MDA contents of mechanically damaged fruits were consistently higher than those of the control and peaked at 25.78 μmol g^-1^, 61% higher than control at 12 days (**Figure 1D**). Under the conditions of HC and LO, the MDA contents exhibited the same up-down pattern (**Figure 1D**). The MDA contents of persimmon fruits under low temperature remained low, and the highest value was only 24 and 38% of the HC and LO treatments, respectively (**Figure 1D**).

### Expression of *DkLOX* Genes in Different Fruit Developmental Stages and Tissues of Persimmon

**FIGURE 2:**
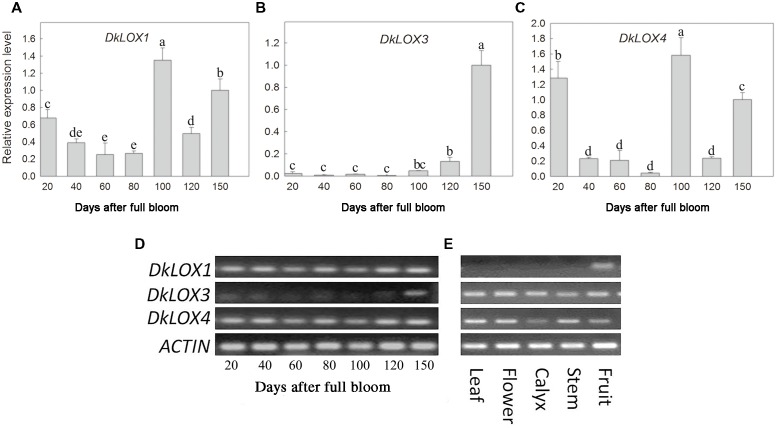
**Expression analysis of *DkLOX1*, *DkLOX3*, and *DkLOX4* of persimmon fruits in different developing stages and tissues with real time quantitative **(A–C)** and semi-quantitative PCR **(D,E)**.** For real time quantitative PCR, data are presented as mean ± standard error from three replicates with three biological repeats, and different letters above the columns indicate significant differences (LSD, *P* < 0.05) between samples across the time-course of the experiment.

The expression patterns of three persimmon *LOX* genes among different developmental stages and tissues were examined (**Figure 2**). In young fruits, *DkLOX1* and *DkLOX4* were expressed at a higher level than *DkLOX3* (**Figure 2D**), and there were two expression peaks of *DkLOX1* and *DkLOX4* at 20 and 100th days after full bloom, respectively (**Figures 2A,C**). The expression of *DkLOX3* was at particularly low levels and increased gradually until the time of harvest (150 days after full bloom; **Figures 2B,D**). In contrast, *DkLOX1* expression was barely detectable in the other tissues, i.e., leaves, flowers, calyces, and stems, while *DkLOX3* and *DkLOX4* were expressed in those tissues (**Figure 2E**).

### Expression Patterns of *DkLOX* Genes in Response to Postharvest Storage Conditions in Mature Persimmon Fruit

**FIGURE 3:**
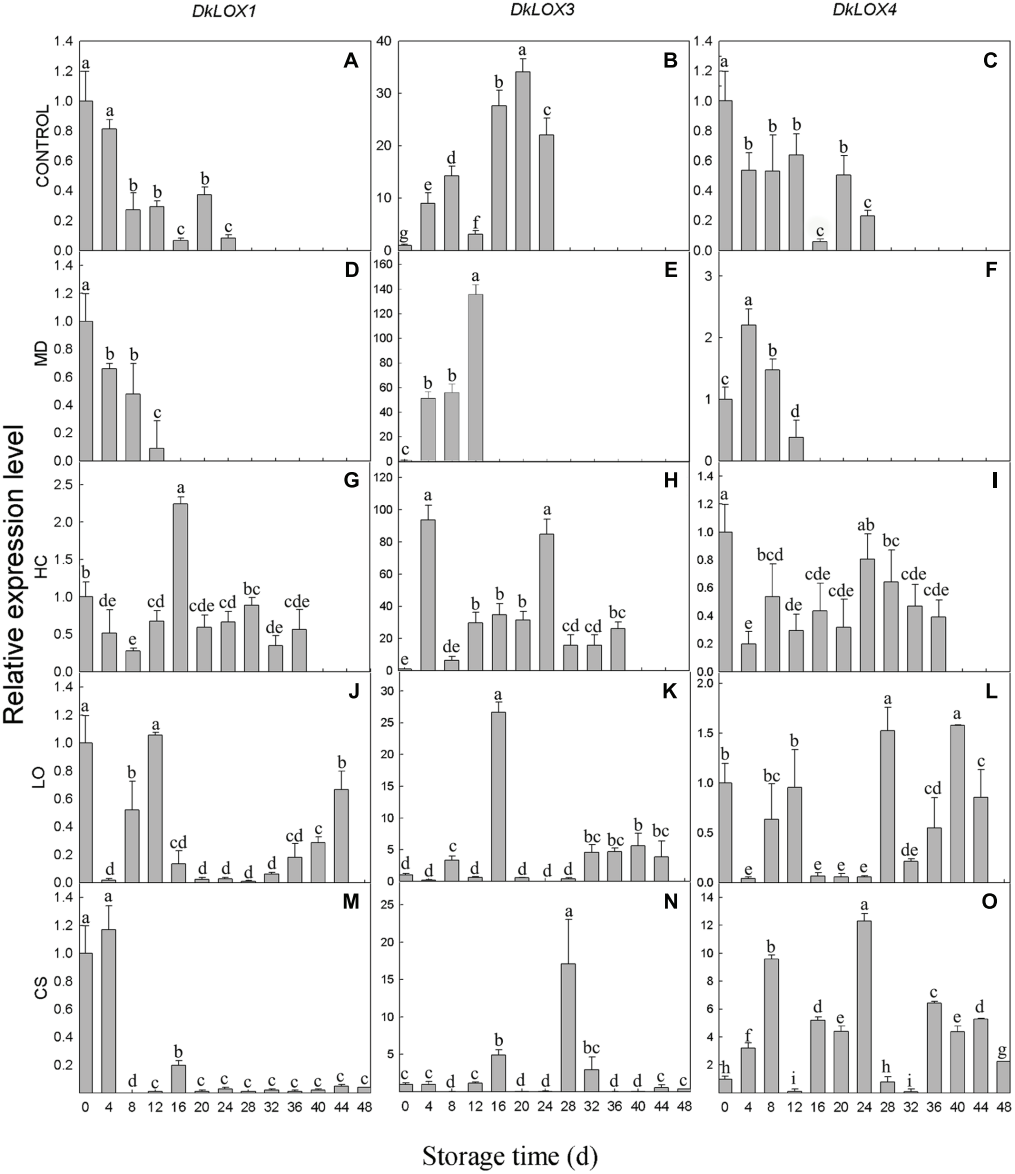
***DkLOX1*, *DkLOX3*, and *DkLOX4* genes expression of persimmon fruits during different postharvest storage conditions.** **(A–C)** Untreated fruits stored at 25°C (CONTROL). **(D–F)** Mechanical damage (MD, 25°C). **(G–I)** High carbon dioxide (HC, 25°C, 6–8% CO_2_). **(J–L)** Low oxygen (LO, 25°C, 2–3% O_2_). **(M–O)** Cold storage (CS, 4°C) . Data are presented as mean ± standard error from three replicates with three biological repeats, and different letters above the columns indicate significant differences (LSD, *P* < 0.05) between samples across the time-course of the experiment.

The expression profiles of three persimmon *LOX* genes were examined in persimmon under different postharvest storage environments (**Figure 3**). The expression of the *DkLOX1* and *DkLOX4* of the control fruits decreased after harvest and displayed low transcript abundances during the whole storage duration (**Figures 3A,C**). Similar patterns were also observed in fruits with mechanical damage and those that were treated under LO and low temperature conditions for *DkLOX1* (**Figures 3D,J,M**), as well as for *DkLOX4* in the fruits stored under HC (**Figure 3I**). In contrast, the expression of *DkLOX1* was up-regulated approximately 2.5-fold of that on the harvest day and then decreased to a low level under HC (**Figure 3G**). Furthermore, unlike the patterns of *DkLOX4* under other conditions, regulation of this gene appeared highly sensitive to low temperature, as it was up-regulated dramatically throughout almost the entire storage duration and peaked on day 24, with approximately 12-fold higher transcript abundances than those on harvest day (**Figures 3B,O**). Compared to *DkLOX1* and *DkLOX4*, the expression of *DkLOX3* was generally higher for all treatments; the transcript abundance of *DkLOX3* in the control fruits increased after harvest and peaked on day 20 approximately 34-fold higher than that from the harvest day before slightly decreasing at the end (**Figure 3B**). The fruits with mechanical damage showed strong *DkLOX3* up-regulation with a peak of 44-fold higher transcript abundance than that the control on day 12 (**Figures 3B,E**). Under HC conditions, there were two peaks in *DkLOX3* expression that were 9- and 4-fold higher than the control on days 4 and 24, respectively (**Figures 3B,H**). In contrast, in the fruits under LO and low temperature conditions, *DkLOX*3 expression was greatly suppressed and maintained at a lower level during the storage period (**Figures 3K,N**).

### Overexpression of *DkLOX3* in Tomato Promotes Fruit Ripening and Softening

**FIGURE 4:**
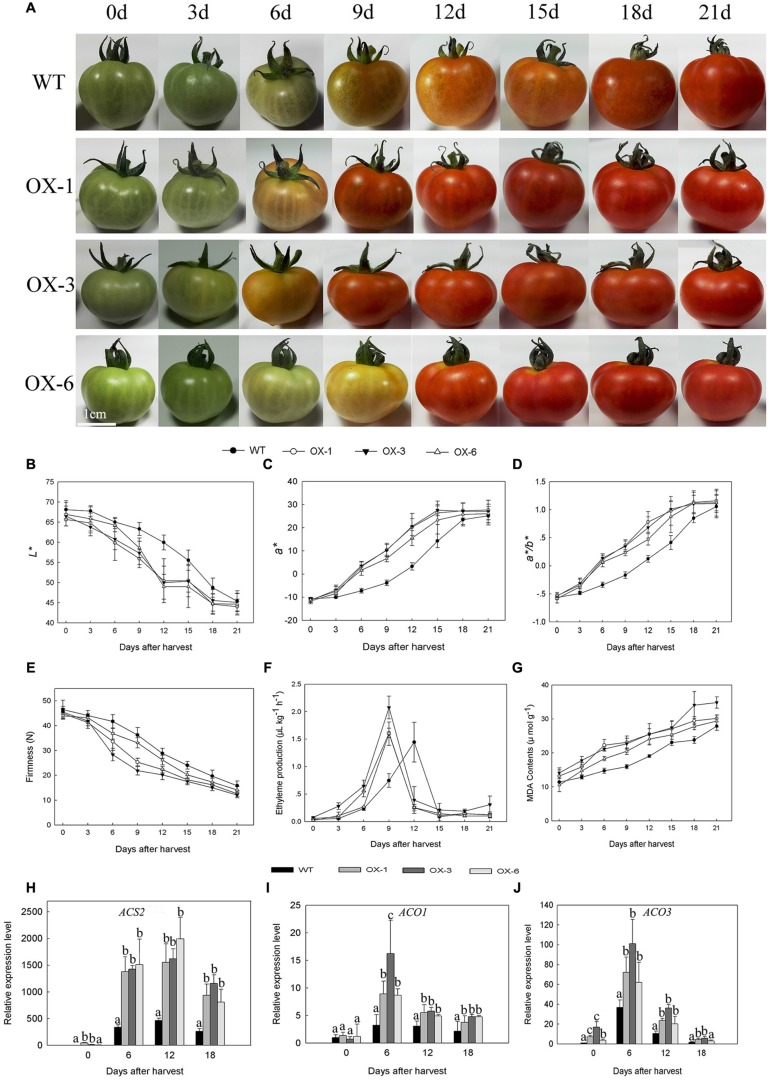
**Phenotype, physiological parameters and related gene expression of wild type (WT) Micro-Tom and three independent transgenic lines (OX-1, OX-3, OX-6) during 21 days’ storage at 25°C after harvested at mature green stage.** **(A)** Fruit phenotype. **(B–D)** Changes in *L^∗^*, *a^∗^*, and *a^∗^*/*b^∗^* color parameters of tomato fruit. **(E–G)** Changes in firmness, ethylene production and MDA contents of fruit. **(H–J)** Ethylene synthesis gene expression level of *ACS2*, *ACO1*, and *ACO3* in different stages of tomato fruit. Data are presented as mean ± standard error from three replicates with three biological repeats, and different letters above the columns indicate significant differences (LSD, *P* < 0.05) between wild type and transgenic lines.

*DkLOX3*-OX transgenic Micro-Tom lines (OX-1, OX-3, OX-6) were obtained to verify whether lipoxygenase is related to fruit ripening and softening. Fruits of transgenic lines and WT were harvested at the mature green stage and stored for 21 days under the same conditions. As the tomato fruits began to turn red, soften and darken, it was observed that transgenic tomato fruits had accelerated maturation and senescence and reduced storability (**Figure 4A**). The maturity was quantitated by *L^∗^*, *a^∗^*, and *a^∗^*/*b^∗^*, with the value of *L^∗^* declining faster, *a^∗^* and *a^∗^*/*b^∗^* values increased more rapidly in transgenic tomato. At 12 days after harvest, *a^∗^* of transgenic fruits was approximately 5–7 folds of WT (**Figures 4B–D**). Meanwhile, the firmness of transgenic fruits decreased faster, MDA contents were always higher, and the appearance of ethylene production peak in transgenic lines occurred 3 days earlier than that of WT (**Figures 4E–G**). In addition, the expression levels of ethylene synthesis related genes were examined. Ethylene biosynthesis genes, *ACS2*, *ACO1*, and *ACO3* were up-regulated to different degrees at all storage periods (**Figures 4H–J**).

### *DkLOX3* Overexpression Promotes Dark-induced *Arabidopsis* Leaf Senescence

**FIGURE 5:**
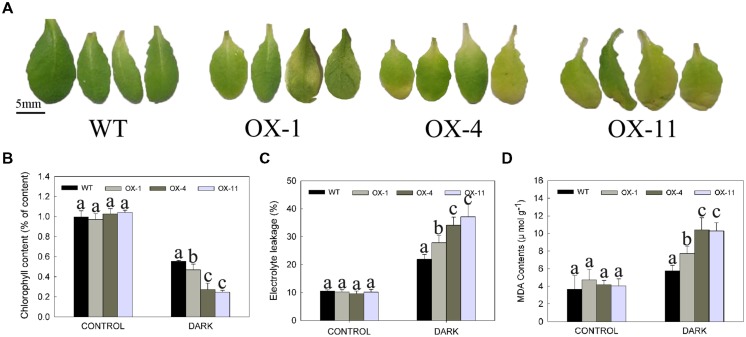
**Dark-induced detached leaves senescence of wild type (WT) and transgenic *Arabidopsis* lines (OX-1, OX-4 and OX-11).** **(A)** Visual appearance of WT and transgenic plants after 4 days in dark. Scale bar = 5mm. **(B)** Chlorophyll content. **(C)** Electrolyte leakage. **(D)** MDA content. Leaf numbers 5 and 6 were from 4-week-old rosettes after sowing. Leaves stored in light served as control. Data are presented as mean ± standard error from three replicates with three biological repeats, and different letters above the columns indicate significant differences (LSD, *P* < 0.05) between wild type and transgenic lines.

**FIGURE 6:**
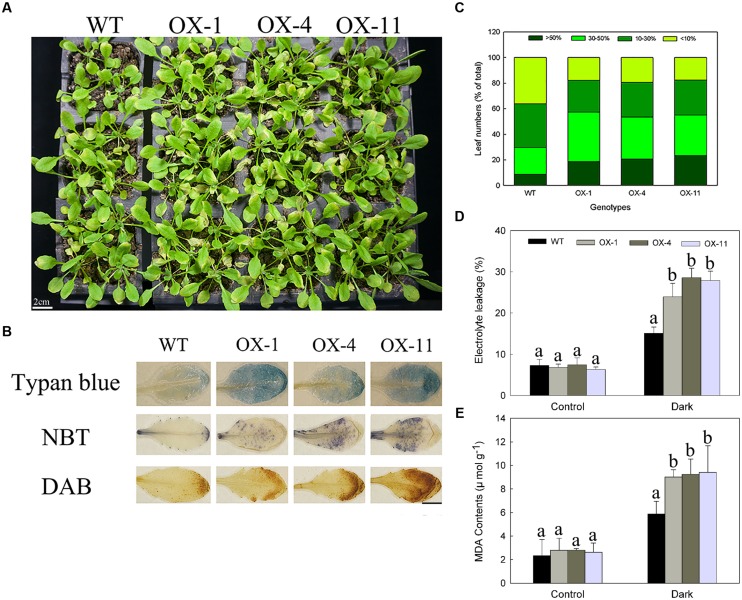
**Dark-induced *Arabidopsis* plants senescence of WT and transgenic lines (OX-1, OX-4, and OX-11).** **(A)** Visual appearance of WT and transgenic plants after 4 days in dark. Scale bar = 2cm. **(B)** Trypan blue staining for cell death, NBT, and DAB staining for detecting levels of O_2_^-^ and H_2_O_2_ production. Scale bar = 5mm. **(C)** The degree of leaves turning yellow. **(D)** Electrolyte leakage. **(E)** MDA content. Four-week-old *Arabidopsis* plants were used for inducing senescence, leaf numbers 3 or 4 were used for histochemical staining. Data are presented as mean ± standard error from three replicates with three biological repeats, and different letters above the columns indicate significant differences (LSD, *P* < 0.05) between wild type and transgenic lines.

The leaf senescence (yellowing) of detached leaves or whole plants of WT and transgenic plants after storage in the dark for 4 days is shown in **Figures 5** and **6**, respectively. Larger areas of yellow could be seen in transgenic leaves compared to WT (**Figure 5A**), indeed, more chlorophyll loss was detected in transgenic leaves (**Figure 5B**). In addition, electrolyte leakage and MDA content were examined as an indicator of lipid peroxidation (Jiang and Huang, 2001; Verslues et al., 2006); transgenic plants exhibited significantly higher levels of electrolyte leakage and MDA content compared with WT plants, especially for OX-4 and OX-11 lines (**Figures 5C,D**).

As with detached leaves, the whole transgenic plants exhibited same accelerated senescence (**Figure 6A**). NBT, DAB, and trypan blue staining of leaf numbers 3 or 4 from the same position showed transgenic plants accumulated more O_2_^-^, H_2_O_2_ and dead cells (**Figure 6B**). After induction in dark for 4 days, the yellow area more than 30% were 57.24, 53.44, and 55.09% for three transgenic lines, respectively, whereas those of WT plants was only 29.79%, which mainly exhibited less than 30% of a single leaf turned yellow (**Figure 6C**). Furthermore, *DkLOX3*-OX *Arabidopsis* plants also had significantly increased electrolyte leakage and MDA contents (**Figures 6D,E**).

### Enhanced Tolerance of *DkLOX3*-OX Transgenic *Arabidopsis* to Osmotic Stress

**FIGURE 7:**
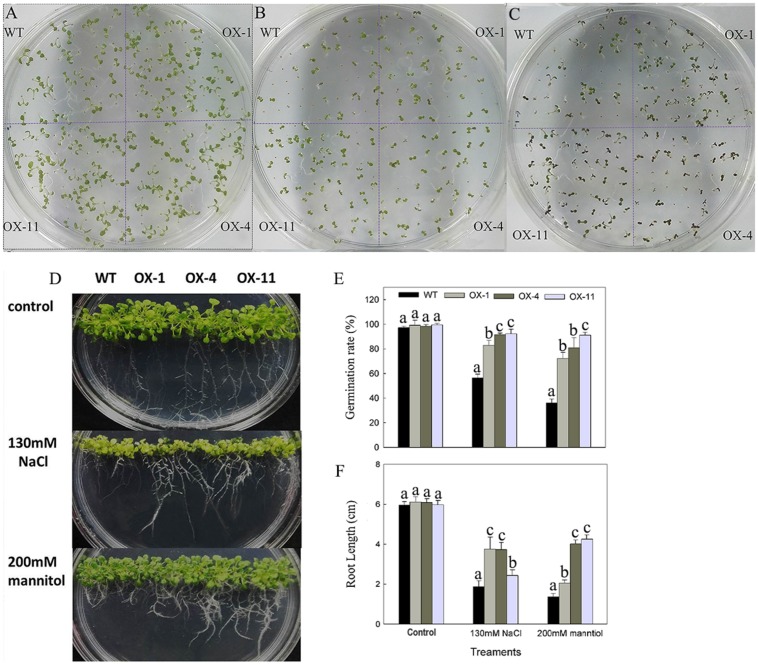
**Phenotype of osmotic stresses on seed germination of WT and transgenic *Arabidopsis* lines (OX-1, OX-4, and OX-11).** **(A–D)** Representative images of seedlings from WT and transgenic lines after being cultivated on one-half MS basal medium **(A)** or one-half MS additional with 130 mM NaCl **(B)** or 200 mM mannitol **(C)** for 8 days, respectively. **(D)** Root growth of WT and transgenic lines for 10 days after cultivated on osmotic medium. **(E)** Germination rate: the percentage of seeds showing radicle emergence after sown. **(F)** Root length. Data are presented as mean ± standard error from three replicates with three biological repeats, and different letters above the columns indicate significant differences (LSD, *P* < 0.05) between wild type and transgenic lines.

To examine the effects of *DkLOX3* overexpression on the germination of seeds under osmotic stress, seeds of the transgenic lines and WT were cultured on one-half MS medium (**Figure 7A**) or one-half MS with additional 130 mM NaCl (**Figure 7B**) and 200 mM mannitol (**Figure 7C**) for 8 days. The germination rates of transgenic seeds and WT were not significantly different on MS basal medium, however, when treated with salt and mannitol stress, the transgenic lines exhibited a 24–55% higher germination rates than that of WT (**Figure 7E**). Furthermore, when cultured vertically for 10 days on 130 mM NaCl or 200 mM mannitol medium, the root length of WT was inhibited more severely (**Figure 7D**), nearly less than half the length of that observed in the transgenic lines (**Figure 7F**).

### Enhanced Tolerance of *DkLOX3*-OX Transgenic *Arabidopsis* to Salt and Drought Stresses

**FIGURE 8:**
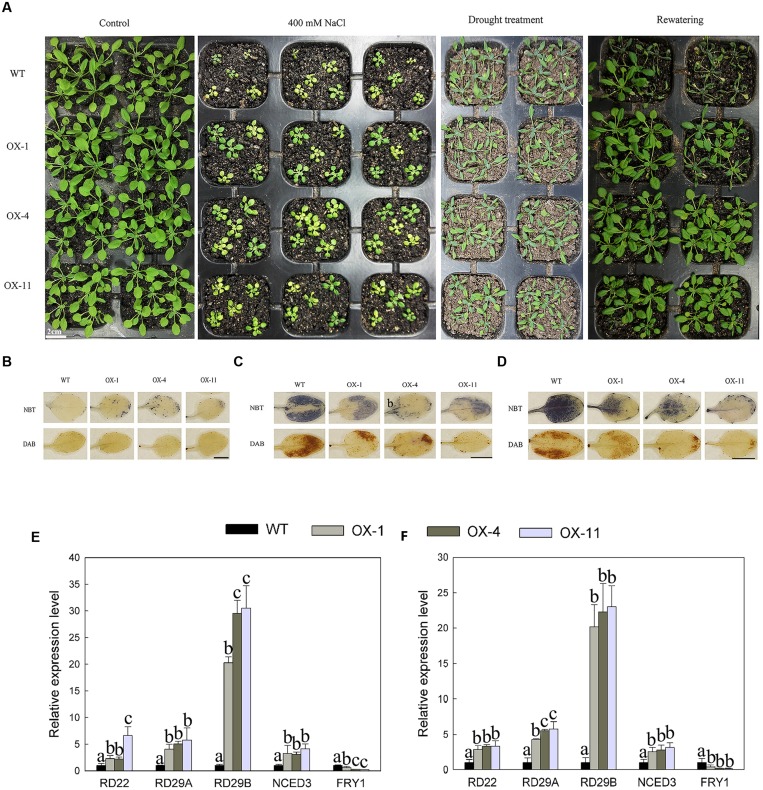
**Effect of salt and drought stresses on seeding growth of WT and three independent transgenic lines (OX-1, OX-4, OX-11).** **(A)** Photographs of WT and transgenic (OX-1, OX-4, and OX-11) plants watered normally and regularly (Control), watered with 400 mM NaCl for 18 days from 8 days old, deprived of water for 10 days of 18-days-old plants and re-watered for 24 h. **(B)** NBT and DAB staining of leaves from control plants watered normally and regularly. Scale bar = 5mm. **(C)** NBT and DAB staining of leaves under salt stress. Leaves were excised at 24 h after treated with 400 mM NaCl with 4-week-old plants. Scale bar = 5mm. **(D)** NBT and DAB staining of leaves under drought stress. Leaves were detached from plants deprived of water for 10 days with 18-days-old plants. Scale bar = 5mm. **(E)** Stress responsive related genes expression from leaves treated with 400 mM NaCl after 24 h. **(F)** Stress responsive related genes expression from leaves deprived of water for 10 days. Data are presented as mean ± standard error from three replicates with three biological repeats, and different letters above the columns indicate significant differences (LSD, *P* < 0.05) between wild type and transgenic lines.

*DkLOX3*-OX *Arabidopsis* plants enhanced tolerance to mannitol and salt-induced osmotic stresses in both seeds germination and root growth. Then further research was performed to establish whether *DkLOX3* overexpression could increase resistance to salt and drought stresses. When 8-day-old seedings were watered with 400 mM NaCl for 18 days, their growth was strongly inhibited with both transgenic and WT plants. However, the suppression of the rosette leaf growth of WT was more serious than that in the transgenic lines (**Figure 8A**). For drought stress, when 18-day-old plants deprived of water for 10 days, the leaves of WT were curly and turned dark green, while those of the transgenic lines were slightly shriveled; 24 h after re-watering, more transgenic plants resumed normal growth than WT (**Figure 8A**). The effect of salt and drought stress on the oxidative burst was determined by detecting O_2_^-^ and H_2_O_2_ accumulation with histochemical staining. Salt and drought stress treatments led to lower accumulation of ROS in transgenic plants (**Figures 8B–D**), and the expression levels of stress-responsive genes were significantly up-regulated with the exception of *FRY1*, which plays a negative role in the ABA pathway (**Figures 8E,F**).

## Discussion

In the present study, it was found three persimmon *LOX* genes were differentially regulated during fruit development and in mature fruits under different postharvest conditions. Among them the expression levels of *DkLOX3* were found to be extremely high in ripening and softening fruits. Furthermore, overexpression of *DkLOX3* showed an acceleration of transgenic tomato ‘Micro-Tom’ fruit ripening and softening and *Arabidopsis* leaf senescence. In addition, overexpression of *DkLOX3* in transgenic *Arabidopsis* enhanced the resistance to osmotic, high salinity, and drought stresses.

For persimmon*, DkLOX1* and *DkLOX4* were expressed in all of the fruit developmental stages, and the expression peaked accompanying by the persimmon growth curve which presented a double “S” (**Figures 2A,C**), possibly indicating that *DkLOX1* and *DkLOX4* were related to fruit growth and development. In contrast to *DkLOX4* which was expressed in all of the organs, *DkLOX1* was not detected in leaves, flowers, calyx, or stems (**Figure 2E**), and its expression profile is consistent with that of potato *LOX1* (Kolomiets et al., 2001). As reported in previous research, potato *LOX1* was specifically expressed in tubers, its mRNA accumulation correlated positively with tuber initiation and growth, and the suppression mutant exhibited a reduced tuber yield, decreased average tuber size, and disruption of tuber formation (Kolomiets et al., 2001).

In this study, *DkLOX3* was expressed at fairly low levels in young fruits (**Figure 2B**), however, its expression was much stronger than that of *DkLOX1* or *DkLOX4* during the ripening and senescence period after harvest (**Figures 3A–C**). According to classification method of Zhang et al. (2006), *DkLOX1* and *DkLOX4* were classified into the first group which had little relationship to ripening, while *DkLOX3* belongs to the other group which plays a positive role in fruit ripening and senescence. In addition, it was found that persimmon fruits treated with MD and HC suffered more rapid ripening and senescence (**Figure 1A**), accompanied by higher LOX activity, and MDA contents (**Figures 1C,D**). This is the same with our qPCR analysis results of the higher expression of *DkLOX3* under these two treatments than others. Simultaneously, the apex of ethylene production was also peaked around the time point of the highest *DkLOX3* transcript levels. Overall, it is suggested that *DkLOX3* probably cooperating with ethylene production play a positive role in fruit ripening and senescence.

Farther verification was done using transgenic *DkLOX3*–OX Micro-Tom to test that whether *DkLOX3* gene plays a role in fruit ripening and softening. *DkLOX3*-OX transgenic Micro-Tom showed more rapid color changes, advanced ethylene peak, increased MDA contents and expression levels of ethylene biosynthesis genes compared to WT (**Figure 4**). In climacteric fruit, ethylene is the dominant trigger for ripening and affects the transcription and translation of many ripening-related genes (Alexander and Grierson, 2002). ACC synthase (ACS) and ACC oxygenase (ACO) are the pivotal regulatory enzymes involved in the synthesis of ethylene. In this study, expression levels of *LeACS2*, *LeACO1*, and *LeACO3* showed a relatively higher expression in transgenic tomato fruits, accompanied by an earlier ethylene production peak (**Figures 4H–J**), while the expression of *ACS1A* and *ACS6* was maintained at a relatively low level (Data not shown). These results agree with previous studies: *LeACS1A* and *LeACS6* are involved in system 1, which is responsible for producing the basal levels in green tomato fruits, *LeACS2* plays an important role in system 2 where ethylene synthesis is initiated and maintained by ethylene-dependent induction at the start of ripening, and antisense inhibition of *LeACS2* could prevent fruit ripening (McMurchie et al., 1972; Oeller et al., 1991). The conversion of ACC to ethylene was carried out by *ACO*, in ripening tomato fruits, where *LeACO1* and *LeACO3* were highly regulated, however, ethylene synthesis is mainly dependent on *LeACO1*, while *LeACO3* expressed at a relatively lower level than *LeACO1* (Alexander and Grierson, 2002). Elevated ethylene biosynthesis genes expression and advanced ethylene peaks also provide evidence for that fruit ripening was promoted, meanwhile, it might be suggested that *DkLOX3* or its products stimulated advanced ethylene synthesis. However, further research is needed to explore the detailed molecular regulation mechanism between lipoxygenase and ethylene biosynthesis pathway.

Furthermore, the relationship of *DkLOX3* and leaf senescence was studied via transgenic *Arabidopsis*. Leaf senescence is easily observed due to the loss of chlorophyll (Quirino et al., 2000), correlated with increases in lipid peroxidation and membrane permeability (Strother, 1988), while MDA and “second messenger of free radicals” were the end products of lipid peroxidation (Ayala et al., 2014). In our research, *DkLOX3*-OX transgenic *Arabidopsis* promoted leaf senescence, accompanied by more chlorophyll degradation, electrolyte leakage, MDA content, cell death and ROS accumulation both in detached leaves and whole plants (**Figures 5** and **6**). These results raised the possibility that overexpression of *DkLOX3* generated enhanced lipid peroxidation and ROS accumulation thus lead to destruction of cell membrane, and then the leaf senescence was exacerbated. However, recent studies suggest that JA does have a role in senescence (Kim et al., 2015). During tomato fruit ripening, the lycopene content was significantly decreased in the fruits of JA-deficient mutants (*spr2* and *def1*), but was enhanced in 35S::*prosystemin* transgenic fruits which had increased JA levels, moreover, the exogenous MeJA significantly promoted lycopene accumulation of the ethylene-insensitive mutant fruits (*Never ripe*, *Nr*; Kim et al., 2015). In *Arabidopsis*, mutants *allene oxide synthase* (*aos*), *oxophytodienoate-reductase* 3 (*opr3*), which have decreased JA levels, and *coronatine insensitive 1* (*coi1*), which is insensitive to JA, exhibit temporal shifts in the onset of natural and dark-induced senescence (He et al., 2002; Castillo and Leon, 2008; Schommer et al., 2008; Danisman et al., 2012). In this regard, it is not clear that whether lipoxygenase, especially 9-lipoxygenase, is involved in JA-induced fruit ripening or leaf senescence.

Exception of inducible expression in persimmon fruit during postharvest senescence period, *DkLOX3* expression was also stimulated by wound and HC treatments (**Figures 3E,H**), indicating that *DkLOX3* have additional function under these abiotic stress conditions. In this respect, *DkLOX3* gene’s functions involved in the response to osmotic, high salinity, and drought stresses were investigated using *DkLOX3*-OX *Arabidopsis* plants. Transgenic *Arabidopsis* exhibited a better tolerance to osmotic stresses with increased germination percentage and better root growth on medium with NaCl or mannitol stresses (**Figure 7**). This is consistant with pepper *CaLOX1*, *CaLOX1-*OX transgenic *Arabidopsis* exhibited a higher germination rate in the presence of mannitol and high salinity (Lim et al., 2015), however, in rice (*Oryza sativa*) endosperm, antisense suppression of *LOX3* gene expression could enhance germinate rate and seed longevity underlying artificial aging or natural aging (Xu et al., 2015).

In addition, transgenic *Arabidopsis* also exhibited enhanced tolerance to drought and high salinity, accompanied by a low level of ROS accumulation and up-regulation of stress responsive genes, including ABA-dependent *RD22*, *RD29A*, *RD29B*, ABA-independent *NCED3*, and downregulation of *FRY1*, which plays a negative role in the ABA-dependent stress signal pathway (**Figures 8E,F**). These results provide the evidence for the possibility that *DkLOX3* or *DkLOX3*-derived products play a positive role in abiotic stresses response by modulating the expression of ABA-dependent and other stress-responsive genes. Several studies also found that products of lipid derivatives were suggested to act as secondary messengers to activate some stress-associated genes and activate the response of the plant to desiccation and salinity (Ingram and Bartels, 1996; Shinozaki and Yamaguchi-Shinozaki, 1997; Ben-Hayyim et al., 2001; Sofo et al., 2004; Ghanem et al., 2012). In *CaLOX1*-OX plants under ABA treatment as well as normal conditions, smaller stomatal aperture was both found and this indicated *CaLOX1* or *CaLOX1*-derived oxylipins played a role in ABA-independent stomatal closure, nevertheless, with drought and salinity treatments, ABA-induced gene expression in *CaLOX1*-OX *Arabidopsis* was enhanced (Lim et al., 2015). All these results demonstrated that the participation of LOX in defense pathways is versatile and complicated.

## Conclusion

*DkLOX3* overexpression accelerated Micro-Tom fruit ripening and *Arabidopsis* leaf senescence with more lipid peroxidation and ROS accumulation. Intriguingly, accumulation of ROS was less in *DkLOX3*-OX transgenic *Arabidopsis* in respond to high salinity and drought stresses. This discrepancy raised the possibility that the diversely biological roles of *LOX* participating in plant physiological process. Due to the apparent functional heterogeneity of *DkLOX3* within different regulatory networks, it is difficult to define the detailed and specific individual pathway to plant senescence and stress response. Furthermore, it would be the next step required to gain a more in-depth knowledge of its roles in these pathways and to elucidate the regulatory mechanisms more precisely.

## Author Contributions

JR and YLH designed the study. YLH, KM, and YH contributed to the experiments. YLH, KM, QB, and BW contributed to the analysis and interpretation of data for the work, YLH and KM wrote original manuscript, JR, BW, JS, and JL revised it critically for important intellectual content, and JR gave the final approval of the version.

## Conflict of Interest Statement

The authors declare that the research was conducted in the absence of any commercial or financial relationships that could be construed as a potential conflict of interest.

## Supplementary Material

**FIGURE S1 | Molecular characterization of wild type (WT) and transgenic plants.** **(A)** Diagram of *CaMV 35S* promoter-*DkLOX3* constructs in the pVBG2307 binary vector. The vector encoding bacterial neomycin phosphotransferase II (*npt II*), which is regulated by *CaMV 35S2* promoter (*35S2*-pro) and 3′-*UTR*, served as selectable marker for transformation. *DkLOX3* was regulated by *CaMV 35S* promoter (*35S-pro*) and 3′-terminator (*nos-ter*). LB and RB indicate left and right T-DNA borders, respectively. **(B)** RT-PCR confirmation of transgenic *Arabidopsis* plants. Lanes: M molecular size marker; V *DkLOX3*-pVBG2307 vector; WT, untransformed wild type; OX-1, OX-4 and OX-11 three independent transgenic lines. **(C,D)** Real time quantitative PCR analysis of *DkLOX3* mRNA levels with 4-week-old transgenic *Arbidopsis* and micro-tom leaves. For real time quantitative PCR, data are presented as mean ± standard error from three replicates with three biological repeats.

## Abbreviations

CS: cold storage
HC: high carbon dioxide
LO: low oxygen
MD: mechnical damage
