# Supplementary material for: The Persimmon 9-lipoxygenase Gene DkLOX3 Plays Positive Roles in Both Promoting Senescence and Enhancing Tolerance to Abiotic Stress
Source: Front Plant Sci. 2015 Dec 10;6:1073. doi: 10.3389/fpls.2015.01073 (PMC4674570; doi:10.3389/fpls.2015.01073)
Supplement: Supplementary file 1 [file Table_1.DOCX]

**Supplemental Table S1** Primers used in real time quantitative and semi-quantitative PCR.

| Primers | Primer sequence (5’-3’) | Products  (bp) | Tm  (^o^C) |
| --- | --- | --- | --- |
| *DkLOX1q* | F: TTCCCAATAGTGTCTCAATC  R: GAACAACAAACACCTTCACC | 119 | 54.3 |
| *DkLOX3q* | F: CACTGCTCTTCCCTACCA  R: CAGAGGGAGAAATCAGTGATACAC | 200 | 56.1 |
| *DkLOX4q* | F: TATCCCAGCACCTCAGACT  R: CGAAAATACTCCCAATAGC | 103 | 55.5 |
| *DkACTIN*  *DkLOX3ORF*  *DkLOX3jd*  *AtACTIN2*  *AtRD22*  *AtRD29A*  *AtRD29B*  *AtNCED3*  *AtFRY1*  *LeACO1*  *LeACO3*  *LeACS2*  *LeUBI3* | F: GGATTCTGGTGATGGTGTTAG  R: CAGCAGTTGTTGTGAAGGAGT  F: GATGCTGCTGCACAAGGT  R: GAGGGAGAAATCAGTGATACAC  F: ATCCGCAATCTTCACCACA  R: ACATCTTCAAATGCCCAAAA  F: TTGTGCTGGATTCTGGTGATGGT  R: CCGCTCTGCTGTTGTGGTGAA  F: GGTTCGGAAGAAGCGGAGAT  R: AGTGGAAACAGCCCTGACGT  F: AAGCAATGAGCATGAGCAAG  R: GGAAGACACGACAGGAAACAC  F: ACGACGGAAACATCGGACT  R: CTTCACCACCAGGAGCAAA  F: TTGATGCTCCAGATTGCTTC  R: GGACCCTATCACGACGACTT  F: CGCAGTAGCACTAGGATTG  R: TTGACACCGAGTTTATTGG  F: ACACGAATGTCACTAGCCTCAT  R: TCCATTGCCTTCATTGCTTCAA  F: TGATCTATCCAGCACCATCTCTAACCT  R: CACATTAGCTTCCATAGCCTTCA  F: CCTCACCATTAGTTCGTTAAGACT  R: CATAGACCAGTTGTCAATACATACG  F: CTACAACATCCAGAAGG  R: TGCAACACAGCGAGCTTAACC | 155  2649  475  167  156  271  181  83  309  197  149  175  143 | 55.0  54.8  56.0  58.0  57.5  58.0  58.0  58.2  56.0  55.0  55.0  55.0  55.0 |

Letters “F” and “R” indicate the forward and reverse primers, respectively.
