# Supplementary figures and images for: The Persimmon 9-lipoxygenase Gene DkLOX3 Plays Positive Roles in Both Promoting Senescence and Enhancing Tolerance to Abiotic Stress
Source: Front Plant Sci. 2015 Dec 10;6:1073. doi: 10.3389/fpls.2015.01073 (PMC4674570; doi:10.3389/fpls.2015.01073)

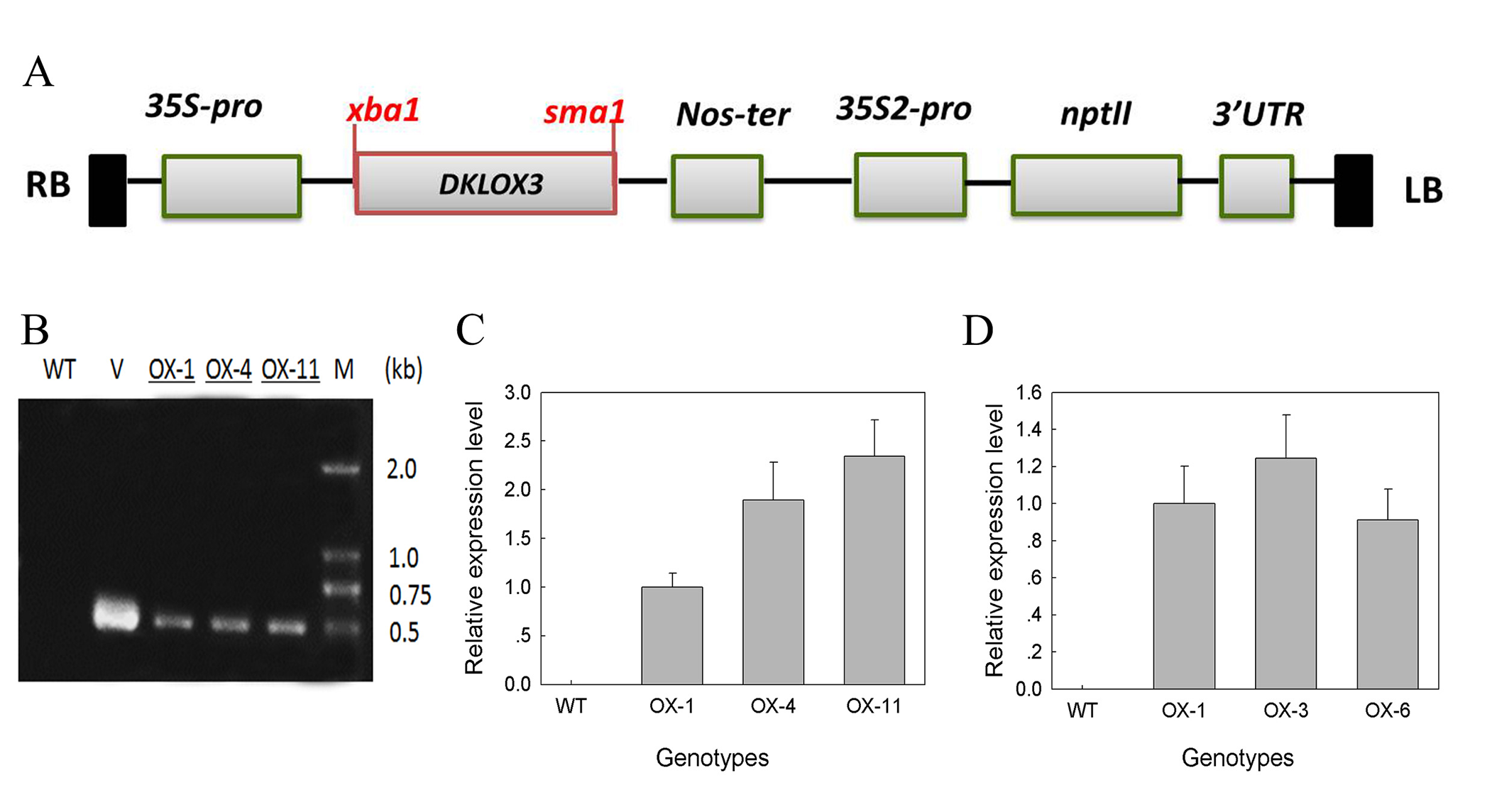

Supplement: Supplementary file 2 [file Image_1.TIF]
